# Australian mental health care practitioners’ construing of non-White and White people: implications for cultural competence and therapeutic alliance

**DOI:** 10.1186/s40359-021-00579-6

**Published:** 2021-05-19

**Authors:** Tinashe Dune, Peter Caputi, Beverly M. Walker, Katarzyna Olcon, Catherine MacPhail, Rubab Firdaus, Jack Thepsourinthone

**Affiliations:** 1 Faculty of Arts, Social Sciences and Humanities, School of Psychology, University of Wollongong, Wollongong, NSW Australia; 2 School of Health Science, Translational Health Research Institute, Diabetes Obesity and Metabolism Translational Research Unit, Western Sydney University, Penrith, NSW Australia; 3 Faculty of Arts, Social Sciences and Humanities, School of Health and Society, University of Wollongong, Wollongong, NSW Australia

**Keywords:** Personal construct psychology, Mental health practitioner, Cultural competence, Therapeutic alliance, Whiteness, Non-White people, White people, Australia

## Abstract

**Background:**

The development of cultural competence is central to the therapeutic alliance with clients from diverse backgrounds. Given that the majority of Australia’s population growth is due to migration, mental health practitioner construing of non-White and White people has a significant role and impact on client engagement.

**Method:**

To examine the impact of mental health practitioner construing on their strategies for cultural competence and the therapeutic alliance, 20 White and non-White mental health practitioners and trainees providing mental health services were purposively sampled and interviewed face-to-face or via videoconferencing. Data was analysed thematically and the impact of construing on practitioner cultural competence and the therapeutic alliance were interpreted using Personal Construct Psychology.

**Results:**

Practitioners demonstrated cultural competence in their acknowledgement of the impact of negative construing of ethnic, cultural, religious, social, racial and linguistic diversity on client wellbeing. Practitioners sought to address these negative impacts on clients by drawing on the client-practitioner relationship to improve the therapeutic alliance.

**Conclusions:**

The results reinforce the need for mental health care workers to develop cultural competence with a focus on developing awareness of the impact of frameworks of Whiteness on the experiences of non-White people. This is central to the development of a therapeutic alliance where clients feel understood and assured that their mental health concerns will not be constructed (and treated) through a framework that constrains both White and non-White people’s opportunities for improved mental health and wellbeing.

## Background

Australia’s cultural, ethnic, religious and linguistic diversity has been changing rapidly, especially within the last 50 years [1]. Over 27% of Australians were born overseas and another 20% have at least one parent born overseas [1]. Further, Australia’s Aboriginal and Torres Strait Islander groups are steadily increasing, from 2% in 1999 to 3.3% in 2016. With its commitment to resettle 20,000 refugees a year since 2012 [2], Australia is a rich and nuanced case study of a migrant-receiving country undergoing rapid change. It can therefore be assumed that with migration having increased significantly within the last decade, the number of non-White clients requiring mental health support has also increased. This diversity requires mental health care systems and practitioners to develop new skills to meet the needs of individuals and groups across cultural, linguistic, racial and ethnic backgrounds.

Mental health professions in Australia have typically been dominated by people from White backgrounds [3, 4]. For instance, the Department of Health [5] reported that out of 26 311 registered psychologists, 73.3% were born in Australia and 0.7% were of Aboriginal and Torres Strait Islander background. There is no clear data on the ethnicity of mental health practitioners beyond Indigeneity in Australia—giving the impression that, besides being Indigenous, the remainder of are White Australians. As such, the number of non-White mental health practitioners is not likely to be representative of the actual diversity in Australia’s population. Much of this is because Australia’s ethnocultural contemporary history of racial dichotomies and hierarchies presents challenges in which all people, and particularly those who are non-White, are subject to the constraints of what researchers refer to as ‘Whiteness’.^1^ Whiteness as social construction was first introduced by W.E.B. Du Bois in 1920 in his essay, *The Souls of White Folk* [7, 8] (p16) where he represents Whiteness as sense of supremacy and entitlement:

But what on earth is whiteness that one should so desire it? Then always, somehow, someway, I am given to understand that whiteness is the ownership of the earth, forever and ever, Amen!

According to Fanon [9] (p128), Whiteness leads the White man to believe himself to be the “predestined master of the world”, or as DiAngelo [10] (p56) recently explained: “Whiteness itself refers to the specific dimensions of racism that serve to elevate White people over people of color”.

These descriptors of Whiteness clarify the nature of the racial dichotomies and hierarchies and, importantly, indicate who reaps benefits from Whiteness and those who do not. Frankenberg [11] defined Whiteness as “a location of structural advantage, of race privileges” and a “standpoint, a place from which White people look at ourselves, at others, and at society” (p. 1). Here then, non-White refers to individuals who are excluded from being beneficiaries of Whiteness as a result of their racial, ethnic, cultural, religious, linguistic, or national identities [12].

It is acknowledged that not all Whites experience the same privileges or to the same degrees, or that Whiteness has always looked the same, or that it lacks impermeable and flexible boundaries. It is further recognised that Whiteness (and the delineators for those who benefit, or are excluded, from it) is constantly under negotiation [13]. Even within this constant state of flux, the role of Whiteness and its consequences endure [14]. It is therefore important to consider and identify the impact of this construct on practitioner construing of non-White (and White) people in Australia.

Importantly, the negative health outcomes and experiences of non-Whites within (mental) health care settings are well documented and related to systems and practitioners that harbor constructions of health and wellbeing based on frameworks of Whiteness [15–18]. To circumvent negative outcomes, it is acknowledged that increasing (mental) health care practitioners’ cultural competency is central to improving client health outcomes [19]. While not directly addressed by standard Australian cultural competence training, this paper posits that explicitly naming and critiquing Whiteness is necessary for practitioners to minimise and reconstruct negative ways of construing and engaging with non-White people within the health care system.

### The role of cultural competence and its impact on the therapeutic alliance

In Australia, little is known regarding mental health workers’ constructions about non-White people. Current understandings of their constructions are implied from the extensively documented negative health outcomes and experiences of minority populations across a range of health care settings (see for example [20]). In general, the core of these experiences are related to health systems and workers harbouring constructions of health and wellbeing based on Anglo-centric and racist frameworks [21]. Researchers and practitioners advocate for increased cultural competency [19] which comprises knowledge, conviction and capacity for action at an individual and organisational level in order to appropriately address the health needs of diverse populations [22]. Eisenbruch [23] also emphasises the skill-based notion of competence that encompasses the system (including health workers) to no less a degree than the clients. As such, cultural competency is defined as “a set of congruent behaviours, attitudes and policies that come together in a system, agency or among professionals and enable that system, agency or those professionals to work effectively in cross-cultural situations” [24]^2^ (p7).

**Fig. 1:**
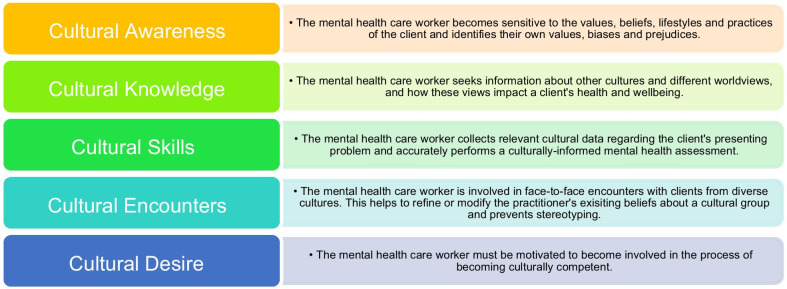
Components of culturally competent mental health care

Building health care workers’ abilities to become self-aware and to reflect on their constructions about others is an important aspect of cultural competency training [19]. According to Campinha-Bacote [26], health professionals, specifically those working in mental health care, need to consistently access training to develop capacity in cultural awareness, knowledge, skill, encounters, and desire.^3^ See Fig. 1, which presents an adaption of De Beer and Chipps [27] cultural competence framework to specifically address the skills required of mental health care workers.

**Fig. 2:**
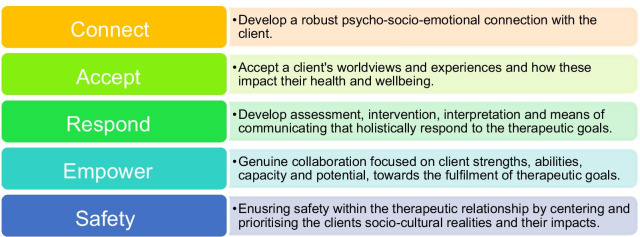
CARES model of therapeutic alliance

By internalising and enacting these components, cultural competence promotes the development and maintenance of a positive therapeutic alliance between practitioners and their clients [19]. Therapeutic alliance is broadly defined as “the collaborative and affective bond between therapist and patient … [and] is an essential element of the therapeutic process” [28] (p438). The therapeutic alliance is characterised by mutual and bi-directional partnerships between practitioners and clients that are dependent on a humanistic healthcare culture [29] and founded on a mutually nurtured psycho-socio-emotional connection [30]. With its origins in early psychoanalytic theories (e.g., [31]), the concept is now a staple of contemporary understandings and evaluations of the therapeutic process. It is well understood that the therapeutic alliance is related to clients’ mental health engagement and outcomes [28]. As such, models of therapeutic alliance often include the following elements: Care, Accept, Respond and Empower (CARE), see for example Sharma and Sargent [32]. In line with the components of cultural competence discussed above and in response to Kirmayer ([25], see footnore 2), Fig. 2 presents a model of therapeutic alliance that was adapted by Escudero et al. [33] to include Safety—a central element for effective engagement with diverse clients.

### Theoretical framework

Personal Construct Psychology (PCP) was developed by George Kelly [34] and is a psychological theory, research methodology and a framework for psychological practice. Originally developed in the 1950s, it has subsequently been extended by many theorists and practitioners [35]. PCP focuses on constructive alternativism; proposing that our experiences of the world, including our understanding of events and people (including ourselves) are open to a variety of interpretations. The particular focus of PCP on individuals as “proactive makers of meaning, as predictors as well as mediators of their psychological reality” [36] (p.39) is preferred over other constructivist approaches. Unlike other approaches (e.g., social constructivism or socioecological theory), PCP was designed to help practitioners understand individual psychology as well as the basis for clinical practice. Consequently, its postulates and principles align well with the concepts of cultural competence and the therapeutic alliance as settings in which practitioners and clients create meaning of one another and then act on their construing within the therapeutic setting. Within the aims of this study, understanding the process of construing is particularly important. Construing is defined as:

an active, ongoing process in which we each constantly try to give meaning to our world and to predict future events by operating rather like a scientist: making hypotheses, testing them out, and if necessary revising them on the basis of the evidence which we collect [37] (p4).

Kelly has further explained that construing is bipolar (e.g., the construct of colour may include black vs white).

Individuals are able to hypothesise what may happen in any given situation through these constructs [34] and “then we test our predictions or anticipations through the behaviours we adopt, similar to scientists engaged in experimentations” [38] (p201). Ideally, in order for a construct to maintain its relevance or applicability, the predicted situation must be validated. Validation occurs when “we see whether any event falls smack on this imaginary point so as to fulfil all of its presupposed conditions” [34] (p86)—our anticipations. This is important to our engagement or avoidance of others given that “our processes—thoughts, feelings and behaviors—operate in a structured manner and are determined by our predictions of the future” [38] (p202).

Existing research reinforces the notion that individuals are in control of their construing and their ability to change their construing of the world towards positive outcomes (a key component of cultural competency and building an effective therapeutic alliance), as demonstrated in Kelly’s theory. In that sense “the aim of [engaging with] personal constructs, put at its most pious, is liberation through understanding” [39] (p201). Using PCP will therefore contribute to a currently limited evidence-base on White and non-White practitioners’ construing and the implications for mental health services in Australia. Given that improved cultural competence and therapeutic alliances depend on revising individual (and societal) constructions, a PCP approach was employed in the design, analysis, and interpretation of the study.

### Significance of the current study

In Australia, there is a dearth of research investigating how mental health practitioners construe and respond to their construing of non-White and White clients. Our recent systematic review [15], which included 5,870 mental health practitioners, demonstrated that constructions about non-White people are intimately linked to workers’ perceived ability to be culturally competent and supportive of an effective therapeutic alliance. The review highlighted that little is known about the impact of construing about non-White people on the strategies that practitioners employ. The review also revealed that training improved cultural competency for the majority of practitioners involved in such programs [40–42]. Across all mental health care professions there remained practitioners however, who rejected or resisted the premise of cultural competency training [19]. It is possible that it may not be cultural competency training itself that some practitioners take issue with, but instead the way in which it is taught and the relatively limited gains which they perceive can come from it. A better understanding of the strategies that Australian practitioners employ in light of their construing of non-White and White people is needed to develop responsive cultural competence training, including strategies for building the therapeutic alliance.

Without research on Australian mental health care practitioners, the bulk of existing research focuses on a nation with a longer history of multiculturalism, viz. the United States [15]. Discussions about race and ethnic dynamics have been taking place in the United States for over 500 years, compared to Australia whose national engagement in such debates is relatively recent (about 150 years) [12, 15, 21, 43, 44]. As such, missing from Australian discourses is a clearer understanding of how mental health care practitioners respond to their own and societal construing of non-White clients within a sociocultural context of Whiteness and what this means for practitioner engagement in culturally competent practice and their ability to effectively support a therapeutic alliance with non-White clients. Given this research gap, the current paper presents findings on the ways in which mental health care practitioners who provide counselling services (fully registered and trainee practitioners) perceive:

1. The impact of their construing of non-White and White people, and Whiteness and non-Whiteness, on Non-White people.
2. The strategies used by practitioners to engage and support clients (non-White and White) while working in the context of their construing and in the context of Whiteness.

#### Research context

A qualitative exploratory approach was utilised to explore the role of practitioner construing in cultural competence and the therapeutic alliance. This qualitative study is part of a larger body of work using a sequential mixed methods design. This larger study commenced with an international systematic literature review (see [15] discussed above) which helped to identify research gaps and informed the foci and questions for the qualitative portion of the study. The findings from the qualitative research have informed the final quantitative portion of the project which used validated survey measures as well as open-ended questions on cultural competence and the therapeutic alliance to assess the potential relationships between the variables identified in the qualitative research with a broader sample of Australian mental health care providers (forthcoming).

## Method

The current paper reports on part of the findings from a qualitative semi-structured interview which was adapted from laddering interview techniques to explore participant construing, superordinate constructs, and strategies to engage and support non-White and White clients [45]. Interviews ranged from 45 to 90 min with the average time being 60 min. Interviews were conducted in the practitioner’s office space or other preferred space (e.g., private room at a university campus or via videoconference). The interviews were audio-recorded and professionally transcribed verbatim. Fourteen interviews were conducted by the first author, TD, a Black Afro-Canadian-Australian. The remaining six were conducted by RF, an Indian permanent resident in Australia. Ethics approval was granted by the university’s Human Ethics Research Committee.

### Interview protocol

In the initial stages of the interview, participants were asked to define two construct poles (e.g., White people and non-White people) and then to select one of those two construct poles (see Table 1). Each participants’ preferred pole was then “laddered”, based on work by Hinkle [46]. This was achieved by continually questioning “why?” after each answer until the participant was no longer able to answer in-depth or with originality. Continuing with this process allows for determining a statement of the values underlying the participant’s construing. It is these identified values that are less likely to be changeable than more subordinate constructs and may have wide ranging implications. For instance, ‘deserving of respect’ may be a subordinate construct to ‘equality’ the more superordinate construct. This laddering process was conducted for three additional ladders on the following constructs: non-Whiteness and Whiteness; how non-White and White people are portrayed; and how non-White and White people are treated (see Table 1).

**Table 1:** Semi-structured interview protocol

Interview questions
1. How would you define “non-White” people?
2. How would you define “White” people?
3. Which definition do you prefer?
4. Why do you prefer the definition you have chosen? (Continue asking “why” questions following the participant’s response until no new responses emerge)
5. Have you ever heard of the term “Whiteness”?
6. What do you think it means?
7. With that in mind, how might you define non-Whiteness?
8. Which definition do you prefer?
9. Why do you prefer the definition you have chosen? (Continue asking “why” questions following the participant’s response until no new responses emerge)
10. How would you say non-White people are portrayed in Australia?
11. How would you say White people are portrayed in Australia?
12. Which portrayal do you prefer?
13. Why is that? (Continue asking “why” questions following the participant’s response until no new responses emerge)
14. How would you say non-White people are treated in Australia?
15. How would you say White people are treated in Australia?
16. Which treatment do you prefer?
17. Why is that? (Continue asking “why” questions following the participant’s response until no new responses emerge)

*Where appropriate, ask the participant what impact their construing have on engaging with non-White and White clients

**Where appropriate, ask the participant what strategies they use to engage with non-White and White clients to support therapeutic outcomes

At the end of the interview, all participants were asked about their experience being interviewed by a non-White person and how this might have influenced their responses or response style

Practitioner cultural competence was explored throughout the interview by asking participants, where appropriate, about the perceived impact of their construing on engaging with non-White and White clients. These questions served as a gauge of practitioner cultural competence in line with the Campinha-Bacote [26] components of cultural competence discussed in the Introduction. Participants were also asked about the strategies they use to engage clients to support therapeutic outcomes amidst their own and social constructions of non-White and White people. This served as a means for exploring practitioners’ engagement with the therapeutic alliance in line with the CARES model described in the Introduction. The findings reflecting practitioners’ construing and pole preferences are discussed elsewhere.^4^ The data on practitioner perceptions of being interviewed by a non-White person will also be discussed in a further separate paper.

### Recruitment and sample

Participants were purposively sampled from a regional university and two metropolitan mental health services through the researchers’ existing networks. The sample, included five each of White practitioners, non-White practitioners, White psychology trainees, and non-White psychology trainees, across a range of other demographic variables [47] Including 10 White, 10 non-White as well as 10 trainees and 10 practitioners ensured opportunities for varied perspectives across racial and experience groups but also data saturation within each group [47]. Purposive sampling also ensured that the sample was diverse and helped to exclude participants who did not engage in clinical mental health care with clients. Participants self-reported a range of clinical employment settings, including public, private, or mixed, their level of experience in terms of both time in practice and engagement with non-White clients, sex, age group, and where they conducted training and practice. Both mental health care trainees and practitioners were included to explore perspectives across diverse levels of experience and training. Participants self-reported on their ethnicity and whether they perceived themselves to be White or non-White. Participant demographics are summarised in Table 2.

**Table 2:** Participant demographics

Demographics	Non-White mental health trainees (n = 5)	White mental health trainees (n = 5)	Non-White mental health practitioners (n = 5)	White mental health practitioners (n = 5)
Sex				
Female	2	4	4	4
Male	3	1	1	1
Ethnicity	Aboriginal Australian (n = 1)	Anglo-Australian (n = 4)	Aboriginal Australian (n = 1)	Anglo-Australian (n = 5)
	Asian (n = 3)	Serbian (n = 1)	Indonesian (n = 1)	British (n = 1)
	Turkish-Muslim (n = 1)		Italian (n = 1)	
			Fijian-Indian-Muslim (n = 1)	
			Zimbabwean (n = 1)	
Languages other than English	Arabic (n = 1)	Serbian (n = 1)	Italian (n = 1)	N/A
	Cantonese (n = 1)		Hindi (n = 1)	
	Mandarin (n = 1)		Shona (n = 1)	
	Teo-Chew (Chinese) (n = 1)			
Age range	19–39 years	20–25 years	33–44 years	28–46 years
Highest level of education	Bachelors (n = 3)	Bachelors (n = 5)	Postgraduate (n = 2)	Postgraduate (n = 5)
	Some tertiary education (n = 1)		Bachelors (n = 3)	
	High school certificate (n = 1)			
Year highest level of education obtained (range)	2005–2019	2014–2016	2007–2016	1998–2016
Country highest level of education obtained	Australia	Australia	Australia	Australia
Citizenship/visa status				
Australian citizen	4	5	5	5
Student visa	1	0	0	0
Role at the time of interview	Psychology intern (n = 4)	Psychology intern (n = 4)	General psychologist (n = 2)	Clinical psychologist (n = 1)
	Youth worker (n = 1)	Youth worker (n = 1)	Health promotion officer (n = 1)	General psychologist (n = 3)
			Social worker (n = 2)	Practice manager (n = 1)
Years practicing psychology (range)	0–1 years	0–1 years	1–14 years	1–21 years
Service type				
Public practice	4	1	5	5
Private practice	1	0	0	3*
No. of non-white clients seen per week (range)	0–6	4–9	1–10 +	4–10 +
Psychology training prepared participant to provide culturally competent services for non-White clients				
Yes	1	3	1	1
No	3	1	4	3
Neutral	1	1	0	1
No. of cultural competence-related professional development since participants last qualification (range)	0	0	7–10 +	0–3

*Three of the White practitioners who worked in the public sector also worked in private practice

### Data analysis and interpretation

Data analysis and interpretation of the interviews included the following steps. In the first step, data underwent conceptual analysis [48] by authors TD and RF in line with the conceptual structures of cultural competence and therapeutic alliance as described in the Introduction. Bender [49] (p82) explains that “conceptual structures are a way of representing knowledge. They can be used to capture knowledge as humans understand it. A conceptual model provides a working strategy, a scheme containing general, major concepts and their interrelations”. Accordingly, conceptual analysis of the interviews included distinguishing terms, identifying the constructs they referred to, and representing these in line with the aforementioned conceptual structures, thus allowing us to make sense of the findings [48]. Myburgh and Tammaro [48] note that conceptual analysis:

is not simply used to discern a set of definitions that are appropriate for the field, but rather the concepts that such definitions might express. The focus is, therefore, of discovering the narratives that are operationalised in this discourse, which is not detectable through the atomising analysis of selected words and phrases, but in those whole-text “explanations” or “procedures” through which professionals run their daily practice.

To support this process, *Quirkos,* a visually intuitive data management software that assists researchers in the coding and analysis of qualitative data [50], was used to organise the participants narratives in line with the constructs explored in this study [51].

In the second step, a lens of PCP processes of transition was used to interpret the results of the above conceptual analysis. According to Kelly [34], “processes” refer to the conglomeration and interaction between our thoughts, feelings, experiences, and behaviours, all of which are determined, not only by society but, also by our efforts to anticipate the world, other people, and ourselves in the moment, in the short-term, and in the long-term. The processes of transition include a Creativity Cycle, Experience Cycle and Decision-making Cycle,^5^ all of which overlap and interrelate in ways that help or hinder us from making sense of the world and how then to react to it, ourselves, and others [34]. Kelly proposes that "the Creativity Cycle is one which starts with loosened construction and terminates with tightened and validated construction" [34] (p7). He further explains that:

loosened construction … sets the stage for creative thinking …. The loosening releases facts, long taken as self-evident, from their conceptual moorings. Once so freed, they may be seen in new aspects hitherto unsuspected, and the creative cycle may get underway [34] (p330).

The Experience Cycle, consists of five phases: anticipation, investment, encounter, confirmation or disconfirmation, and constructive revision. Finally, the Decision Making Cycle, consists of first circumspection, pre-emption, and control. These overlapping, cyclical, and parallel cycles can make it hard for even the most culturally proficient person to holistically and continuously dismantle, reorganise, and reconstrue constructs, constructions and construing of themselves and others.

These dynamic processes link conceptual analysis to transition given that “concepts correspond to ideas, and are conditional and provisional, rather than fixed: they are not neutral, as they correspond to and are motivated by paradigms and ideologies” [52] (p70). While conceptual analysis helped to identify processes, as articulated by the participants, in line with the conceptual structures of cultural competence and therapeutic alliance, interpreting these through Kelly’s processes of transition helped to make sense of participants’ thoughts, feelings, experiences, and behaviours in relation to working with non-White clients. This framework for analysis and interpretation helped the authors to reflect on the impact of practitioner construing, as well as the strategies used to support cultural competence and the therapeutic alliance. This process also assisted in the development of recommendations for research, teaching, and practice, which are presented in the Discussion section.

## Results

Conceptual analysis of participant responses helped to identify narratives that reflected components of the Campinha-Bacote [26] cultural competence conceptual model and elements of the CARES therapeutic alliance conceptual model. Quotes that were most representative of participant narratives were used to illustrate where practitioners’ perspectives and experiences aligned (or not) with the components and elements within the aforementioned conceptual models. The impacts of practitioner construing of Whiteness on cultural competence are presented within *Enacting Cultural Competence in the Context of Whiteness* and practitioner strategies to improve the therapeutic alliance are presented within *Knowing Me, Knowing You…Despite Whiteness*.

### Enacting cultural competence in the context of Whiteness

As noted in the introduction, central components of cultural competence to be addressed within the context of Whiteness are cultural awareness, cultural knowledge, cultural skill, cultural encounters, and cultural desire (see Fig. 1 in the Introduction). Representative participant quotes are included to highlight the role of each component in their re/construing.

#### Cultural awareness

An integral starting point is the ability for health care providers to be aware of and understand the impact of their construing, and subsequent behaviours, on their clients. Several practitioners demonstrated cultural awareness in their analysis of terms like “culturally and linguistically diverse” and its limitations. For example, an Anglo practitioner stated:

Why do we say culturally linguistically diverse? It is, apparently, more culturally sensitive. It’s not as branding. We’re not defining someone by the colour of their skin … I think it’s exactly what we’re doing … It’s like a circular term, to say the same thing without saying that thing. Why don’t we want to say it? Why don’t we want to say non-White? There are connotations of power imbalance.

This practitioner views these terms as meaningless and highlights that they are likely to perpetuate ignorance about the impact of Whiteness and the practitioners’ role in erasing race by being afraid to name it. While it may be easier to identify who one is not (e.g., ‘the Other’), identifying oneself as White and identifying the implications of that Whiteness are needed for White practitioners to develop cultural awareness. Another Anglo practitioner explained:

It means that our language, our power structures, our social hierarchies have been formed on the basis of being White, again, there’s that real historical impact in terms of colonisation …With that history, that’s what we’ve built on and so that’s what we exist with today, here.

This practitioner highlights the importance of having information about the past to better engage in cultural competence. As such, the ability to recognise the power structures and roots of Whiteness and to then articulate its impact on non-White people requires practitioners to have a adequate levels of historical and cultural knowledge.

#### Cultural knowledge

The majority of non-White and White practitioners (70% of all participants) felt that their cultural knowledge was inadequate. Perhaps due to a lack of adequate cultural competence training, White practitioners’ failure, or resistance, to ask questions about diversity were perceived by non-White practitioners as barriers to White practitioners’ engagement with clients. For instance, the Aboriginal practitioner who ran cultural competence training noted the conundrum faced by White professionals who may resist asking questions:

I used to do cultural training and stuff like that, there was no right or wrong questions. If you don’t ask the questions you are not going to grow as a professional, as a White professional, you are going to constantly have these barriers between you and Aboriginal culture. Ask it, it’s not offensive; it’s more offensive when you don’t ask because we know when you’re not asking.

For this practitioner, it was important for others to confront Whiteness and its impacts by engaging in difficult or uncomfortable discussions or asking seemingly silly questions. However, being able to do so requires practitioners to both have and seek opportunities to develop cultural skill.

#### Cultural skill

Cultural skill is concomitant with mental health practice skills, with both ensuring accurate assessment, diagnosis, and intervention within a client-centred framework. An Anglo trainee describes the development of both cultural and psychological skills to ensure a nuanced assessment of an older Aboriginal man:

One of them was for an assessment, so learning assessment for an Aboriginal man, and untangling, the complication was untangling for dyslexia assessment … He was old and lived through the Stolen generation, so trying to entangle what would be dyslexia, what would be a psychological disorder, what’s pathological, what’s gone wrong versus what was systematic kind of racism; what’s happening at a society and it would be understandable with this reading and you know and kind of there wasn’t a guide book for that.

This trainee continued to expound on their pursuit of opportunities for repeated construing and reconstruing, through a range of pathways including supervision and additional research. This process demonstrates the importance of analysing the needs of the client within a historical context of racial oppression. Without this approach, the trainee may have reproduced the oppressive practices of Whiteness by pathologising the client’s symptoms.

#### Cultural encounters

The development of cultural competence requires practitioners to directly engage with people different from themselves and to actively seek opportunities to learn from diverse people. Practitioners indicated that the more cultural encounters they had, the less comfortable they felt with stereotyping others. This was not limited to White identifying practitioners and trainees. A Muslim Turkish trainee explained:

I don’t like to generalise a lot … I just can’t, because then you meet somebody and you’re so surprised about their knowledge, you’re so mind blown you’re like “I should never be stereotyping” and I’ve had that happen to me quite a few times when I get so caught up in my judgements so to speak and my stereotyping and it just slaps me in the face going no, don’t do that. So I’m just so hesitant to do that nowadays … let them present themselves before I make a judgement.

As noted by this trainee, reconstruing one’s perceptions and understandings of clients requires practitioners to consciously critique their own construing and reconstrue their role in the therapeutic setting. This is not always easy and therefore requires that the practitioner have cultural desire to engage in this often uncomfortable and confronting process.

#### Cultural desire

Learning from cultural encounters and engaging with cultural difference and the potential for discomfort requires practitioners to desire the challenge and the benefits of working towards cultural competence. The African practitioner used the following analogies to describe her desire to be culturally competent:

For me I can think of it in the sense of I want to have done something significant in my career … I want to live to my full potential, but I feel like to live to your full potential doesn’t have to be just in terms of career things, so even if it’s mothering that you raise your child well, you raise them to be good citizens, you raise them to be, you know, honest, decent people. If it’s friendship that you become the best friend that you can. It’s just doing the best that you can in any of the roles that you embody … like philosophers would say it’s always a project, always in the making.

These analogies highlight that cultural desire comes from a craving to understand and to construe differently such that one’s view of the system is challenged and revised. In doing so changes in construing then manifest into action and changes in behaviour. One Anglo practitioner explained:

I like this [engaging in the interview about Whiteness], because you learn a lot and, to be honest, I think there’s so much of this that needs to be brought into these discussions …. I just find it intriguing. I enjoy meeting and connecting with other people, that’s a reason why I do the job. That’s the big picture and I just try and understand different ways of people seeing the world. A different way they do the things, from my own and from the current thing and just learn a bit about it.

With a broadened world-view, that goes beyond Whiteness as the privileged default, practitioners can develop a robust therapeutic alliance with non-White clients. This allows practitioners to considers the client as a part of a larger system that influences their experiences, perceptions, and, therefore, their mental health. An Asian trainee explained:

Considering and then thinking beyond Whiteness allows me to gain a better understanding and appreciation of the culture and the systems here, so the education system, the legal system, how everything works and runs here so I don’t—I’m not disrespecting anyone or clashing, having a clash of ideologies and values, because I feel like if you can understand how things work or understand how someone behaves or thinks then that’s where you can be more respectful. Because normally acts of disrespect come when you don’t understand or don’t have a knowledge of their culture or their person, so I feel like it’s important for me to understand so I can prepare in advance in a sense.

This desire to engage with systemic and broadened perspectives allows for the development of a therapeutic alliance that is safe and respectful. In such a space clients and practitioners can develop competencies in construing and re-construing themselves, each other, and the world in ways that improve (mental) health and wellbeing outcomes.

### Knowing me, knowing you…despite Whiteness

Therapeutic alliance is reliant on the client and the practitioner acting as partners in the therapeutic process. However, mutual partnership cannot be achieved if the practitioner fails to understand individual non-White clients as products (and potentially agents) within a system—in this case a White system. The CARES model (see Fig. 2 in the Introduction) for therapeutic alliance seeks to guide practitioners towards this goal. Despite the impact of Whiteness, participants described how they were able to *Connect*, *Accept*, *Respond*, *Empower,* and ensure *Safety* with their non-White clients.

#### Connect

A foundational element of therapeutic alliance is the practitioner’s ability to develop a connection with the client that demonstrates respect instead of judgement or stereotyping. An Anglo practitioner described their strategy to connect with non-White people in the following way:

I’m thinking about [non-White] friends and just may be opening a bit of dialogue. It’s often not a topic of conversation. I guess the more you look, the more you know or if there is gaps you’re looking and talking at it. Asking more about, you know, how we can connect in a deeper level with [the] other and how we can share those things. There may be times when they [non-White people] need something different from me.

Seeking deep connections with different others ensures that practitioners can develop more meaningful alliances with their non-White clients and encourages construing revisions within the client and the practitioner. An Italian practitioner explained how this kind of connection improves future individual and community health and wellbeing outcomes:

If we commit to really knowing ourselves and others we will have a better society, again, we’ll have more happy, compassionate people that I think will be able to give more to themselves, to give more to their families, give more to their friends. But, more importantly give more to children who are our future.

This long-term and communal sense of health and wellbeing requires an acknowledgement of the limits to our knowledge and perceptions to fuel the desire for connection with diverse people and contexts. However, this kind of openness to connection requires acceptance of uncertainty and being comfortable engaging with the unknown.

#### Accept

Mental health practitioners are trained to thrive within the unknown, such that they continue hypothesising (construing and reconstruing) about their client throughout their entire relationship. Such training ensures that practitioners are able to accept a client’s worldview and experiences and to actively seek to understand how these impact on the client’s health and wellbeing. An African practitioner describes how manifesting such acceptance can support the therapeutic alliance:

[working with diverse clients] it’s important because otherwise—because it gets me to accept that there are things that are beyond my control that will have huge implications for my life. That in fact a lot of the things that happen in my life are beyond my control. The only thing that is certain that I have control over is how I react to things or how I feel about things. It’s important to be comfortable with uncertainty, to live with uncertainty because it is going to happen now and again in the future …. I have to be able to learn with those things because they are beyond my control.

The African practitioner reflects on learning about acceptance through the therapeutic alliance, as a means to help them to develop increased self-awareness and knowledge about the impact of social systems on herself and her client. This type of acceptance creates a mutual space in which the power of the practitioner and client become more balanced, with the client teaching the practitioner and the practitioner developing their capacity to appropriately respond to that individual client within the context of the system.

#### Respond

Responding to working with different others can be challenging when the practitioners’ value system is at odds with that of their client. Non-White practitioners seemed to experience this more intensely, or more consciously, given that they have always had to navigate the consequences of being non-White in a White world. When working with White clients they have had to recalibrate their “cultural compass” in order to respond in ways that recognise that White people are also imbued in a White system that also has significant impacts for them too. An Aboriginal practitioner reflected:

I suppose White people also have languages, religions, practices and traditions and I suppose their value system and their morals are formed by this cultural compass that you’re constantly carrying around with you all the time. It’s like a lens that you see the world in.

Interviewer: Cultural compass, I like that. So it’s the way they see life, engage in life?

Aboriginal practitioner: Yeah. And people, like relationships, make decisions, experience the world and experience other people, particularly in psychology the way that you formulate someone, just a simple case formulation, your interpretation of a theory, your interpretation of a technique and an intervention.

This practitioner references “relationship” as a central point through which people “make decisions” and “experience the world and other people”. This is central to construing and the therapeutic alliance as a place for reconstruction and action. Notably, the practitioner acknowledges that the “way you formulate someone” and “your interpretation” are in response to one’s cultural compass. Being aware of this cultural compass and its role in case formulation and “interpretation of a technique and an intervention” is central to responding to clients in their context—in this case, the context of being non-White, working with a White person, and amidst a socio-cultural context of hierarchical Whiteness. The Italian practitioner explained the impact of this context on their response to disadvantage:

I think if you are poor and White you have it really tough. But, if you are White with money, then again you’ve got more opportunities, better access. Yes. So, I think if you’re poor and White then you fall into this multicultural kind of community. You kind of like move over because you’re getting maybe the same services and accessibly as what non-Whites would be getting. You have to adapt for that, right? Like, we can’t just say ‘yeah forget about what is missing and sort it yourself because they are White.’ There is more to consider, more to do.

The dynamism presented in the above quote requires the practitioner to “adapt” and therefore respond in ways that acknowledge diversity in client experiences in the context of Whiteness. Such adaptation requires that both parties, especially the client, feels empowered to be an agent in that process and not just an object, as Whiteness would often relegate them.

#### Empower

Empowerment within the therapeutic alliance requires genuine collaboration focused on client strengths, abilities, capacity, and potential toward the fulfilment of therapeutic goals. Practitioners supported empowerment of non-White clients by drawing on various sources and, most importantly, the client themselves. The White British practitioner explains how she takes an empowering approach to their work with non-White clients:

I would take it [culture] into account when I’m designing a treatment plan rather than making assumptions that, this has evidence so therefore it will be successful for my entire client base – CBT – No! For example, so in my individual interactions I’m getting to know people and their cultural context then I can, ask them at their level to individualise I can seek information from research based on country of origin or culture of origin or particular language or religious practices and then show them you know ‘this is what I’m planning I know you don’t necessarily have an understanding about psychology in general but you do have an understanding of you so how does this sound?’ I do this anyway but open up discussion I’m going you know please give me feedback I’m open to feedback. I welcome complaints and feedback; I welcome you bringing a family member; I welcome you contacting us outside the session if you need something other than what you’re getting.

By centring culture and feedback, this practitioner’s strategy to empower supports the client’s ability to be an agent within the therapeutic setting and to define the model of mental health care that works best for them. Importantly, the client is drawn into the practitioner’s learning process and is provided with opportunities to correct the practitioner if they are misunderstanding the cultural context and/or developing strategies that are not aligned with the clients’ values or perspectives. In this way, the therapeutic alliance can become a safe place for mental health improvements aligned with the client’s goals.

#### Safety

The development of interventions that deviate from a one-size-fits-all approach supports the therapeutic alliance and setting as a safe space where clients do not have to act White in order to have their needs met. The ability to be oneself within the therapeutic setting encourages the practitioner and the client to consistently transition their construing in ways that promote wellbeing and progress—both clinically and systemically. An Anglo practitioner describes this process with an Asian client with social anxiety:

Interviewer: Similarly, what does your role, as a White person, perhaps interacting with a non-White person, what does that do in the room? What happens to the therapeutic alliance in that sense?

Anglo practitioner: I have been conscious of it, some of the different clients that I have …. She’s a young Cambodian girl with social anxiety and she was talking the other day about being the only Asian on the field. She’s playing sport and how difficult it is as the only Asian on the field. She’s saying she just doesn’t like communicating with White people at all, because it’s difficult … This girl has so many communication difficulties, but in part of my thinking, and because I’m White, I’ve thought, you know what, I should keep her [as a client] because I think this will help her build relationships outside of what she is comfortable with.

Interviewer: Relationships with White people?

Anglo practitioner: Yes.

Interviewer: That in itself is therapeutic

Anglo practitioner: That’s informed some of my thinking … creating a safe place for her to develop her communication skills.

For most non-White people, communicating about the frustrations caused by Whiteness with a White person is, at best, a nerve-racking experience. Such an experience could easily be panic-inducing for a non-White person with social anxiety. However, the fact that such a discussion could happen with this practitioner indicates that the therapeutic alliance was strong enough to allow the client to be an agent and communicate about her discomforts in a safe setting. Here, the practitioner used the relationship as a therapeutic tool—supporting both the objective and outcome of a strong therapeutic alliance. As explained by an Aboriginal trainee, a robust therapeutic alliance cannot be faked or fabricated. Such true alliance with a client is one where the client and practitioner feel safe enough to be open, vulnerable, and open with each other in ways that breed empathy and understanding in the same way that reciprocal and mutual love cannot be faked:

Aboriginal trainee: If you disagree then disagree but stick by it so that you know who you are and you are authentic to that, because if you are not authentic to yourself and your beliefs and stuff how can you be authentic to ours, or empathic, at least empathic to ours.

Interviewer: Right. So based on that you can’t fake it. It’s not possible then?

Aboriginal trainee: I don’t think you can. Like in a relationship with someone, we talked about therapy alliance you can’t fake loving someone, so you can’t fake having a therapeutic alliance on a cultural level, you can’t fake it.

## Discussion

The current study presents the findings from interviews with White and non-White mental health practitioners and trainees providing psychology services. The study explored how clinicians construed: (1) the impact of constructions of non-White and White people and Whiteness and non-Whiteness on Non-White people, and (2) the strategies used by practitioners to engage and support clients (non-White and White) while working in the context of Whiteness. These inquiries were used to qualitatively examine the role of practitioner construing in cultural competence and the therapeutic alliance.

No major differences were found in terms of sex, age, areas of practice or education. However, non-White and White practitioners as well as Non-White trainees produced more strategies for cultural competence and therapeutic alliance than did White trainees. This is likely because non-White practitioners and trainees may already have the fundamental background knowledge required to be culturally competent which practice only amplifies. This is corroborated by other research [40–42] in which non-White trainee cultural competency scores start off higher than White practitioners and then level out post training. As found by Bitney [20], it is likely that White practitioners also produced more strategies than their trainee counterparts as a result of their experience working with non-White people, given that cultural encounters are a core element towards developing cultural competency [26].

Notably, only one White practitioner (a British self-identified expatriate) and all non-White practitioners had engaged in further cultural competence training outside of their formal education. In an American study on social workers’ perceived cultural competence near equal proportions of African American and Anglo-American participants had attended between one and three cultural competence continuing professional development courses since their formative qualification [53, 54]. The findings from the current study may therefore be unique to the Australian context where pressure to engage with cultural competence is relatively recent. Further it may speak to the fact that, in the balance of priorities, continued engagement in the challenging task of cultural competence is not high on the list for mental health practitioners’ continuing professional development. Our current quantitative research with a broader Australian cohort assesses the generalizability of this assertion.

### Making sense of practitioner construing

Campinha-Bacote [26] notes that the process of continuously developing cultural competence requires practitioners to demonstrate cultural desire. This desire was characterised by practitioners’ ability to consistently seek out, engage in, and to tolerate the disintegration and transitioning of their cultural constructs—central aspects of Kelly’s Process of Transition. The findings of this study demonstrate that practitioners were consistently engaged in the Creativity Cycle as they expressed being in a relatively constant state of constructive openness, despite the pervasive constraints of Whiteness that bear down on us all. Despite this, it is acknowledged and well documented that the development of, and continuous engagement in, cultural competence is challenging given the requirement to confront and change one’s construct systems to align with the needs of the client [13, 18, 55–58]. Participants demonstrated the Experience Cycle by drawing on cultural competencies like skill, knowledge, and, especially, encounters to challenge themselves to reconstrue, revise, and redevelop the strategies they used to develop and support the therapeutic alliance with non-White clients. In this study, practitioners demonstrated the utility of the Decision Making Cycle by actively seeking out opportunities for cultural awareness that assisted them in working collaboratively with non-White clients to develop and then use the therapeutic alliance as a tool to support the client in taking an empowered and more agentic role in their wellbeing. This is, of course, a challenge given that society and the health systems therein are constructed to align with biomedical models based on Eurocentric ways of knowing and behaving. In the absence of a society and systems that adapt at the same speed as diversity occurs, practitioners are tasked with finding common ground with their clients.

This common ground is the therapeutic alliance which is characterised by mutual partnerships between practitioners and clients, dependent on a humanistic healthcare culture [29]. This is not to say that practitioners should be blind to difference and treat everyone the same—a position widely contested by scholars, researchers and practitioners alike [13, 58, 59]. Strategies used by practitioners in this study provided real-life examples of each element within the CARES model and offered both the client and the practitioner a space where difference and the re/construing of oneself and others could be explored within the safety of an interpersonal interaction occurring at a discrete point in time. Thinking about the process of cultural competence in this way can potentially alleviate many mental health care practitioners’ resistance to cultural competence training and construing transitions [60]. With cultural desire as motivation; when done repeatedly; and with a variety of clients, cultural competence increases and more impactful therapeutic relationships can be established. This is a reward for both the client and the practitioner for whom improved mental health outcomes are the goal and to which the therapeutic alliance is positively correlated [59].

### Implications for theory, research, teaching, and practice

It is acknowledged that Kelly’s Personal Construct Psychology was based on his work with White people for working with White people and within the framework of Whiteness. Despite its Anglocentrism, the relevance of his theory has been tested and utilised in a diverse range of non-White and marginalised population settings [38, 61–64]. Importantly, it offers a way for practitioners, who are trained to focus and “work on” the individual, a framework to expand their understanding of the systems and influences in which psychological practice occurs for the client and the practitioner. While other systems theories provide a robust understanding of various levels of influence on individuals, they do not provide an operational framework from which practitioners can develop practical processes to support the development of cultural competence and the therapeutic alliance. This study confirms the utility of PCP as an effective means of understanding and contextualising the challenges practitioners may experience when working with non-White people and their potential resistance to developing cultural competence and culture-informed therapeutic alliances as a result of Whiteness and its rigidity.

Further research is therefore needed into the impact of practitioner construing of diverse populations on culturally competent practice to help mental health care practitioners and systems better respond to the needs of diverse populations with the development of cultural awareness, knowledge, and skills in mind. To better understand why this might be, a scoping review exploring the contents of cultural competence training might be an appropriate starting point. Further, perspectives from psychology educators and students can be sought to determine what works and what does not within an Australian context. This would help to identify gaps in content with the goal of developing a best practice model for training and assessment.

Based on the findings of this study, a key element of training should include an acknowledgement of the role of practitioner construct systems and processes of construing that result in resistance to training and construing transition. This should be done initially and regularly across psychology training programs. The Australian Psychological Society [65] acknowledges this gap on their website stating that “one of the challenges for the future will be working towards embedding relevant cultural awareness and curriculum content across 40 higher education providers”. Addressing this gap may improve cultural desire and circumvent restrictions in learning, development, and client health outcomes as a result of psychological resistance [66].

This study therefore reiterates the role of the therapeutic alliance as a prime setting for practitioners to deconstruct and reconstruct their construing about “others” and the frameworks through which construing are made. Such a process supports the development of a therapeutic alliance where clients feel understood and assured that their mental health concerns will not primarily be constructed (and treated) through Whiteness—a framework that constrains both White and non-White people’s opportunities for improved mental health and wellbeing.

## Limitations

A few limitations should be recognised in interpreting the study results. First, generalisability of findings is limited given the small sample size and the ability within this sample to account for diversity. The qualitative sampling methods are, however, appropriate for exploring the unique and rich perspectives required to fulfil the aims of the study and fulfil the imperative in qualitative research for trustworthiness [67]. Second, despite their vast diversity, the locations (Illawarra Region and South West Sydney of New South Wales) from which participants were recruited may not reflect the experiences of other practitioners across Australia. To expand the representativeness of this exploration, the authors are currently conducting further research using validated quantitative methods to investigate practitioners’ construing, perceived cultural competence, and role of the therapeutic alliance across diverse practitioner and client populations. Finally, while the adapted laddering interview technique helped to elucidate practitioners’ constructs and construing, the forced construct of non-White and White, and Whiteness and non-Whiteness, does restrict the participants’ opportunities to delineate and define what these concepts mean and how, if at all, they are related to one another. Further investigations could use different interview techniques to assess the trustworthiness of the findings from this study.

## Conclusion

In this study, 20 White and non-White mental health practitioners and trainees providing psychology services were purposively sampled and interviewed using a semi-structured interview that drew on the laddering technique. This study aimed to provide evidence to advance Australia’s capacity to holistically support its increasingly multicultural population through genuine acceptance, and integration, of diversity. Using Personal Construct Psychology to interpret the findings in relation to processes of transition helped to explain the development of practitioners’ cultural competence. It also helped to understand the quandary of the resistance faced by practitioners to cultural competence training and their concomitant cultural desire which allowed them to acknowledge the impact of Whiteness on (non)White clients. Personal Construct Psychology highlights the need for practitioners to, not only tolerate but, desire and, therefore, seek out opportunities to consistently loosen and reconstruct their construing about themselves and others. While the classroom is often considered the place for the development of cultural competence, the authors suggest that the associated skills are best honed within the safety of the therapeutic alliance.

Interactions with clients become opportunities for the development of the therapeutic alliance, but also a space where both the practitioner and the client can safely loosen their construing of one another. The benefit, for both parties, is the opportunity to deliver and engage in treatment with full recognition of the impact of Whiteness but without having to be bound by it. The relationship can be the site for and source of reconstruing of the other. This is in line with Personal Construct Psychology, specifically Faidley and Leitner [68] (p72), who noted that “we interact out of, and at the same time create mutually constructed meaning [within the interpersonal process]”. With an increasingly diverse population, the challenge for all people is to welcome interpersonal processes and the concomitant discomfort of engaging with re/constructions that confront the ways that we make sense of the world—providing avenues for constructive alternativism. The findings from this investigation are therefore significant for the development of cultural competency training, practice, and theory that reflects the learning needs of mental health practitioners.

## Data Availability

Upon request, all relevant raw data will be freely available to any scientist wishing to use them for non-commercial purposes and without breaching participant confidentiality.

## Abbreviations

CARE: Care, accept, respond, empower
CARES: Care, accept, respond, empower, safety
PCP: Personal construct psychology

## Footnotes

1 The capitalisation of the ‘W’ in White and non-White is one of contentious scholarly debate. A capital W is used in this paper in line with *The Diversity Style Guide* (2019) to acknowledge the racialisation and concomitant characterisation of ethnic, religious, cultural and linguistic affiliation in terms of proper nouns used to construct identities and not simply to inaccurately describe phenotypical traits.

2 It is acknowledged that these somewhat simplistic, skill-based and practitioner-focused constructions of cultural competence are limited in their recognition of the impact of racial dichotomies and hierarchies that result in the perpetuation of disadvantage and devaluation of non-White frameworks, perspectives or people.

3 These constructs are explained in depth in Campinha-Bacote J. The process of cultural competence in the delivery of healthcare services: A model of care. Journal of Transcultural Nursing, 2002:13(3):181–184.

4 Dune, T., Caputi, P., Walker, B.M., Firdaus, R., Olcon, K., MacPhail, C. & Thepsourinthone, J. (in press). Construing Non-White and White Clients: Mental Health Practitioners’ Superordinate Constructs related to Whiteness and Non-Whiteness in Australia. *Journal of Constructivist Psychology*.

5 While outside of the scope of this paper, full descriptions of these three cycles in the process of construing are described in Kelly G. The psychology of personal constructs. New York: W. W. Norton & Company; 1955.
